# Cognitive graph transformer: integrating emotional semantics and lexical concepts for computational personality assessment

**DOI:** 10.1038/s41598-026-42640-7

**Published:** 2026-05-04

**Authors:** Shahryar Salmani Bajestani, Seyyed Ali Zendehbad, Mohammad Mahdi Khalilzadeh, Marjan Vatanpour, Elias Mazrooei Rad

**Affiliations:** 1 Department of Biomedical Engineering, Mashhad Branch, Islamic Azad University, Mashhad, Iran; 2 Faculty of Engineering & Technology, University of Mazandaran, Babolsar, Iran; 3 Department of Biomedical Engineering, Khayyam University, Mashhad, Iran; 4 Biomedical Engineering Department, Khavaran Institute of Higher Education, Mashhad, Iran

**Keywords:** Attention mechanism, Cognitive modeling, Graph neural network, Natural language processing, Personality trait detection, Social networks, Engineering, Mathematics and computing

## Abstract

The widespread use of social media has facilitated the recognition of personality from user-generated online content. While numerous applications exist across diverse domains, such as recommender systems, most current studies focus on superficial, statistical, and explicit user content, thereby neglecting latent knowledge. In this study, we propose a method for uncovering latent psycholinguistic understanding at deeper levels of user data to enhance personality prediction through natural language processing. The proposed approach leverages fine-tuning of a domain-specific Bidirectional Encoder Representations from Transformers (BERT) model for sentence-level feature extraction and enriches the output by incorporating emotional information. This process emphasizes salient words through a single-way attention mechanism. Our single-way attention mechanism propagates information from highlighted words to the overall extracted knowledge. Subsequently, using the embeddings from the previous stage as node features, we construct a graph. A dynamic, task-oriented learning approach is then employed to determine the graph edges, using a neural network to connect different pairs of nodes. Finally, a graph neural network is combined with a classifier to predict personality traits. Experimental results demonstrate the effectiveness of the proposed model, achieving 80.27% accuracy on the Essays dataset and outperforming existing approaches. Furthermore, several ablation studies were conducted to investigate the impact of various components and parameters of the proposed architecture.

## Introduction

With the advancement of communication technologies and the emergence of social networks, human interactions have entered a new phase. Social networks not only provide a platform for interpersonal communication but also serve as a medium for expressing emotions, thoughts, and daily experiences^1^. These large-scale interactions have generated massive volumes of textual data, which serve as a valuable resource for analyzing user behavior and personality traits. For instance, Kabir et al.^2^ identified the severity of depression by analyzing users’ tweets. The Big Five personality model is among the most validated and widely applied psychological frameworks for analyzing and understanding human personality. This model comprises five core dimensions: Extraversion, Agreeableness, Conscientiousness, Neuroticism, and Openness to Experience^3^. Each of these dimensions explains different aspects of an individual’s behavior and emotional state, and collectively they provide a comprehensive representation of personality. Identifying the personality types of social media users through text analysis has emerged as a novel and appealing research domain, with a wide range of potential applications, including targeted marketing, user experience enhancement, consumer behavior prediction, recommender system development, and even security threat detection^4^. For example, marketing companies can design more effective advertising campaigns by gaining a deeper understanding of user personalities, while social platforms can recommend more suitable content to their users. The primary objective of this paper is to present a novel and effective method for predicting users’ Big Five personality traits on social networks based on textual analysis. To this end, we first review related work, then introduce our proposed text-based approach for personality recognition, and subsequently compare it with existing methods. Finally, we present the results of our implementation and evaluation of the proposed model, discussing the associated challenges and limitations. By integrating psychological knowledge with advanced data analysis techniques, this study aims to deepen the understanding of user personality on social media and foster improved interactions on these platforms^5^. To offer a more specific conceptual map of existing research, we rearrange the relevant work into three thematic strands. The former is traditional machine learning and Linguistic Inquiry Word Count (LIWC)-based methods, which tend to rely on predetermined lexical classes but are often unable to reveal interactions between linguistic indicators and cognitive mechanisms. The second strand is concerned with deep learning and sequential models, such as Recurrent Neural Networks (RNNs) and transformer-based models, which enhance representational capacity, but continue to view text as a linear sequence. The third thread is the study of graph-based and attention-based methods, in which recent sentiment and emotion analysis work has explored using SenticNet, semantic, or multimodal graphs to model relational information more effectively. Examples Representative Exemplary Representations Dual-graph designs Dual-graph designs represent a category of graph-based models that consider both semantic and affective information when inferring fine-grained sentiment. Multimodal graph designs are another category of graph-based models that use both semantic and affective data to make fine-grained sentiment predictions. Personality prediction methods can generally be categorized into two groups. The first approach involves extracting linguistic features based on statistical attributes and combining them with classical machine learning algorithms, such as Support Vector Machines (SVMs). These approaches attempt to infer personality traits by analyzing linguistic features such as word counts, sentence lengths, the use of specific vocabulary, and their frequencies. The second approach employs deep learning to capture latent knowledge from textual data, followed by personality classification. Deep neural networks, including Convolutional Neural Networks (CNNs) and RNNs, demonstrate strong capabilities for extracting complex features and predicting personality traits. Traditional machine learning and deep learning approaches have shown that language-based models can effectively and accurately identify personality traits of social media users. However, many studies primarily focus on statistical word embeddings such as GloVe and Word2Vec^6,7^. Early studies on automatic personality prediction focused on linguistic features, such as the LIWC tool, which uses machine learning to map words to psychologically relevant categories and count their frequencies to predict personality traits^8^. For example, Salsabila et al.^9^ demonstrated that personality prediction accuracy on Twitter could be improved through LIWC, while Tandra et al.^10^ utilized LIWC-derived features for predicting Big Five traits on the MyPersonality dataset using machine learning techniques. Furthermore, subsequent research highlighted that extracting affect-related knowledge from text can further improve the accuracy of personality recognition frameworks^11,12^. Recent developments in sentiment and emotion analysis increasingly rely on graph-based architectures, underscoring the significance of relational modeling beyond linear text sequences. The dual-graph mechanisms studied in previous works combine fine-grained sentic data, a local convolutional network with co-attention, and multimodal architectures that combine visual/affective features with textual data to predict aspects^13–18^. These models, including the domain of semantic–sentic hybrid graphs with the addition of affective knowledge to adaptive multimodal sentiment tasks based on multifunctional GNNs, demonstrate an expansion of the tendency toward structured representations that capture delicate emotional and contextual dependencies. Although these studies are beneficial for understanding the usefulness of graph reasoning, they, however, pay more attention to sentic, semantic, or multimodal relations; thus, cognitive structures and psychologically based cues are unlikely to be represented in the graph formulation. Since 2014, with the advent of end-to-end deep learning architectures, many studies have explored optimized feature extraction for personality prediction. Majumder et al.^19^ proposed a deep learning method based on one-dimensional convolutions for personality recognition from text, while Dutta et al.^20^ designed a semi-supervised embedding clustering model that simultaneously learned cluster assignments and feature representations to infer personality traits from the Kaggle Personality dataset. Following this trend, pre-trained models such as GloVe and BERT have been increasingly employed for personality prediction tasks^21^. In line with this, El-Demerdash et al.^22^ reported competitive accuracy on the MyPersonality and Essays datasets by proposing a transfer learning framework that leverages pre-trained models, such as ELMo^23^, ULMFiT^24^, and BERT^21^. Building on these advancements and the introduction of attention mechanisms, researchers have focused on extracting semantic features from text. Ramzani et al.^25^ developed an attention-based graph knowledge network for the automatic prediction of personality. Graph Neural Networks (GNNs) can learn node-level feature representations and solve relational tasks using input structural information^26^. New models integrate both sequential and structural information through graph-driven architectures to improve predictive accuracy and interpretability, illustrating how structured dependency modeling can reveal deeper patterns in complex data^27^.Similarly, social recommendation research has shown that graph neural networks enhanced with hybrid similarity metrics can more effectively capture latent user–item interactions, leading to notable gains in link-prediction performance^28^. Beyond these areas, hybrid computational frameworks have also been applied to evaluate institutional efficiency and productivity, demonstrating the value of structured analytical methods for informed decision-making in organizational contexts^29^. Along this line, Zhou et al.^30^ proposed a personality prediction model based on GNNs, constructed upon psycholinguistic knowledge. To address the challenge of encoding long documents with sequential or hierarchical models, which may introduce errors into personality prediction, Yang et al.^31^ proposed a deep graph convolutional network that employs a learning-based graph construction approach. Additionally, they introduced TrigNet, a hybrid architecture that incorporates a triplet graph network and a BERT-based graph transformer, leveraging structural psychological knowledge from LIWC for personality recognition^32^. Similarly, study^33^ proposed Semi-PerGCN, a semi-supervised heterogeneous graph model for personality classification, which integrates LIWC features to enhance graph construction and imposes constraints on shared parameters of GNNs for personality recognition. Although classical machine learning and deep learning methods have demonstrated efficiency in identifying personality traits, several limitations remain. For instance, most embedding techniques rely heavily on statistical word representations (e.g., Word2Vec, GloVe), which may pose challenges for personality prediction. To address this, attention-based approaches such as BERT are employed to recover contextual word embeddings. Moreover, many studies have incorporated LIWC into their architectures for personality classification; however, because of its limited vocabulary coverage, LIWC often fails to generalize well across diverse input data. A large body of research has utilized pre-trained models, such as BERT, to capture text semantics; however, it typically focuses solely on semantic features or combines them with statistical features without leveraging domain-specific knowledge. Finally, integrating textual features with latent affective information at the word level to enhance predictive performance poses additional challenges, which we aim to address in our proposed method by systematically addressing each component. To address these limitations, we frame our study around the following central problem: current linguistic analysis models struggle to capture the interplay between surface-level lexical cues and deeper cognitive structures that shape psychological expression in text. Building on this gap, we formulate three guiding research questions: (1) How can cognitive and linguistic cues be jointly modeled to represent psychological processes in natural language better? (2) Can graph-based cognitive representations enhance the interpretability and predictive strength of transformer architectures? (3) To what extent does integrating cognitive structures improve downstream performance across diverse psychological text-analysis tasks? These questions motivate the development of the proposed Cognitive Graph Transformer and guide the empirical investigations that follow.

## Materials and methods

Given an input text sequence X, the task is to predict a set of psychological trait scores Y, where each member represents the degree to which the text expresses a specific personality dimension. The objective of the model is to learn a mapping X→Y that leverages lexical, cognitive, and affective cues embedded within the text. To achieve this, the Cognitive Graph Transformer constructs a cognitively enriched graph from the input sequence, applies Single-Way Attention to emphasize salient cues, and generates representations that support accurate prediction of the target trait scores. The classification of personality traits can be formally expressed as follows:

Given a document $D=\{S\_1,S\_2,\dots,S\_N\}$ for all subjects, where each sentence $S\_i=\{W\_1,W\_2,\dots,W\_M\}$ consists of $M$words; our objective is to identify the personality traits $P=\{P\_1,P\_2,\dots,P\_t\}$ of the subject based on the latent knowledge embedded in the document $D$.

**Fig. 1:**
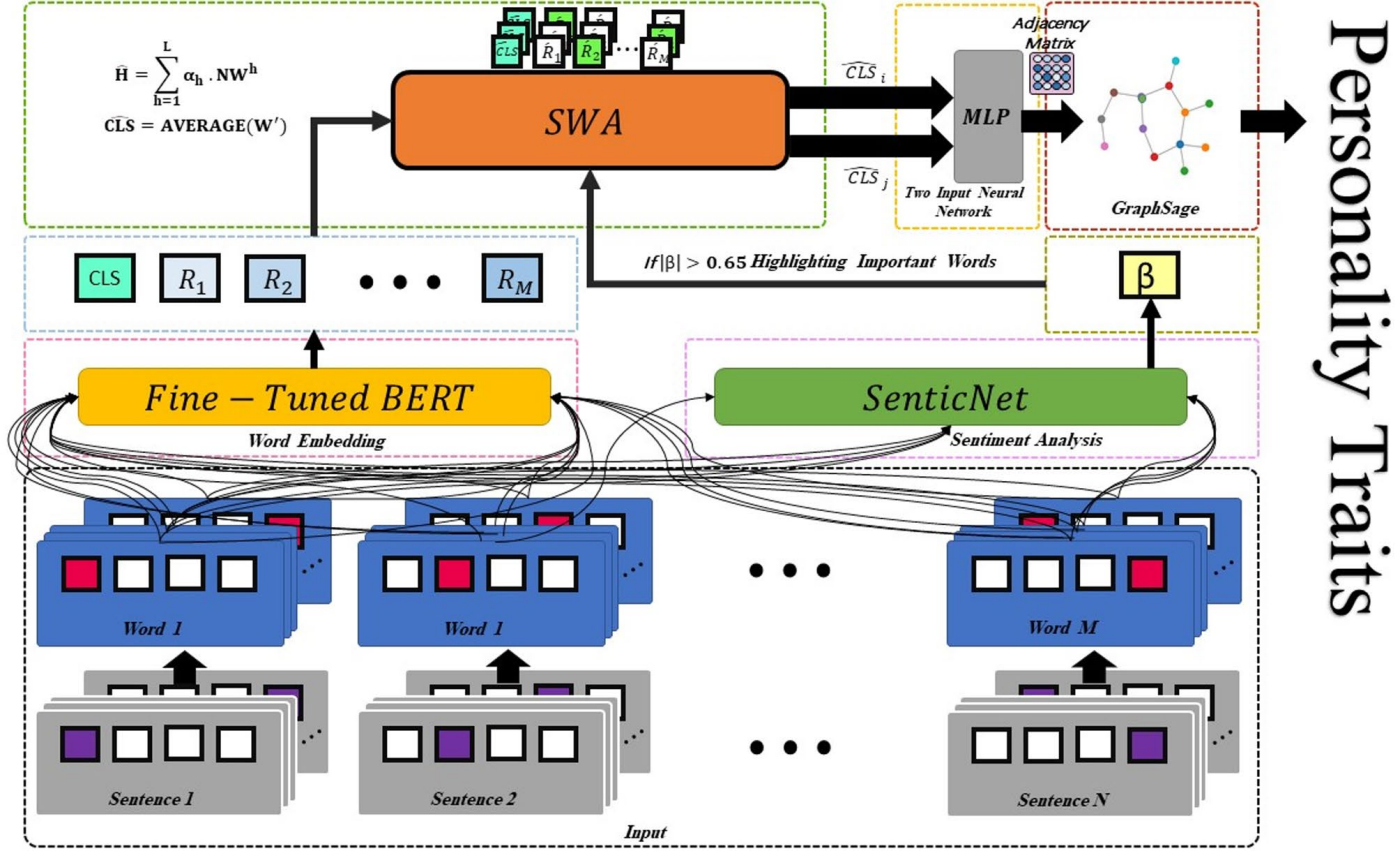
Overall architecture of the proposed method.

The overall structure of Cognitive Graph Transformer is illustrated in Fig. 1. In the following, we present the details of the proposed architecture. A high-level overview of the key components is summarized as follows:

- Input: Providing task-relevant and valid input representations for both the BERT model and the Sentic network.
- Domain-specific fine-tuning of BERT.
- Feature extraction using the fine-tuned BERT model.
- Highlighting emotionally salient and important words through the sentic network.
- Application of a single-way attention mechanism to enrich the CLS embedding in a single stage.
- Propagation of highlighted word importance to neighboring words, followed by averaging across all embeddings, including CLS, to construct the enhanced representation $\widehat{CLS}$.
- Graph construction:
  - Using $\widehat{CLS}$ representations as nodes within a graph neural network.
  - Building edges between node pairs through a dynamic, task-oriented approach.
- GraphSAGE construction: Building a GraphSAGE-based network to aggregate information across neighboring nodes.
- Output: Personality trait classification based on the final node embeddings.
- Fine-tuning RoBERTa: Applying domain-specific fine-tuning of the RoBERTa model to enhance contextualized text representations.

### Input representation

As the input layer, the initial embeddings of each word are computed using BERT. However, to obtain domain-specific word embeddings, the base BERT model is fine-tuned on task-relevant datasets. To this end, we fine-tune the pre-trained BERT model, unfreezing all 12 layers and updating their weights during training for the personality classification task. This allows us to obtain more refined contextual embeddings for words. Specifically, we use a learning rate of 0.0001, a batch size of 32, and the AdamW optimizer. After fine-tuning, each tokenized word can be represented as a vector. Each input sentence can thus be expressed as a matrix of size M×F, where M denotes the number of tokens (approximately equal to the number of words in the sentence) and F = 768 is the hidden dimension of BERT embeddings. Inspired by^34^, we treat each sample as a multi-sentence input associated with a subject’s personality. Here, K denotes the number of inputs for each subject. Accordingly, if multiple tweets or essays are available for a subject, each is treated as a separate input instance and processed individually by BERT. In subsequent stages, after computing the updated CLS embeddings, the outputs corresponding to the K inputs are averaged to form the node features representing a subject. The BERT architecture for classification tasks comprises 12 hidden layers and 12 attention heads, with a hidden size of 768. The semantic representation of the entire input sentence is captured in the CLS vector, derived from the final hidden layer of BERT, which aggregates the global sentence-level information. Additionally, each input token generates its own contextualized embedding, which, along with the CLS vector, is retained for further processing. As illustrated in Fig. 1, the architecture produces both the CLS embedding, representing the overall meaning of the sentence, and the contextual embeddings of individual tokens.

### Highlighting important words

The Sentic network incorporates affective features that provide conceptual information about words and enable the extraction of each word’s emotional polarity. Following^34^, which highlights the relationship between personality and emotion, the polarity scores obtained from the Sentic Lexicon are exploited for two primary purposes. First, polarity scores highlight words that have a substantial impact on sentence semantics. For each token in the input text, we query SenticNet to obtain its associated polarity value and concept-level affective attributes. If a token appears in SenticNet, its polarity score is retrieved directly; otherwise, we use a fallback procedure that attempts to match its lemma or the nearest concept. A thresholding approach with θ = 0.65 is used to identify these salient words. Second, polarity scores can be used indirectly to construct the edges of graph G, as described in subsequent sections. At this stage, we extract the polarity score of each word in a sentence and highlight essential words using the thresholding strategy, as depicted in Fig. 1. Consequently, words are divided into two categories: essential words and ordinary words. Given that the total number of encoded tokens in a sentence is $W\in\{1,2,\dots,M\}$ we define a subset $Q\in\{1,2,\dots,J\}$ containing only the critical words, where ${\text{0 < J}} \le {\mathrm{M}}$. The remaining ordinary words are denoted as ${\text{0 < J}} \le {\mathrm{M}}$.

### Single-way attention mechanism

Hidden semantic relationships exist between essential words and their neighboring ordinary words within a text. Since we employ BERT for word encoding, positional information is already incorporated into token representations through positional encoding, eliminating the need for additional positional embeddings. To capture the interactive relationships between highly influential words and their surrounding ordinary words, we introduce an attention mechanism that enhances the encoded vectors from the previous stage and implicitly integrates polarity information. In this way, meaningful words that carry richer personality-related information can influence the embeddings of their neighboring words. By restricting the information flow so that essential words influence ordinary ones but not the reverse, we preserve the integrity of the salient cues while still enabling them to guide the contextual representation. Inspired by^35^, we design a Single-Way Attention (SWA) mechanism, in which the influence of essential words on ordinary neighboring words is formally defined as follows:

1 $${e}_{iw-nw}={Avr\left({Q}^{j}\right)}^{T}.{W}_{iw-nw}.{NW}^{h}$$

2 $${\alpha}_{h}=\frac{\mathrm{e}\mathrm{x}\mathrm{p}\left({e}_{iw-nw}\right)}{\sum_{h=1}^{L}\mathrm{e}\mathrm{x}\mathrm{p}\left({e}_{iw-nw}\right)}$$

3 $$\widehat{H}=\sum_{h=1}^{L}{\alpha}_{h}.{NW}^{h}$$

Let ${e}_{in}$ denote the function for identifying relevant words, ${W}_{in}$​ a trainable matrix, $Avr\left({Q}^{j}\right)$ the element-wise average of essential words, ${NW}^{h}$ the vector extracted for ordinary words from BERT, ${\alpha}_{h}$ the attention weight corresponding to each ordinary word, and $\widehat{H}\in\{1,2,...,\mathrm{L}=\mathrm{M}-\mathrm{J}\}$ represent the updated embeddings of ordinary words after incorporating the influence of essential words. The new word set is defined as $W^{\prime }  = QU\hat{H} \in \{ 1,2, \ldots ,M\}$. Once the influence of all words on ordinary words is integrated, the single-way attention mechanism is applied: first, the importance of the key words is propagated to the ordinary words, and then the average of all embeddings, including CLS, is computed to obtain $\widehat{CLS}$. As previously mentioned, CLS represents the overall semantic meaning of a sentence. By applying the attention mechanism and averaging along with the CLS vector, it can be concluded that the latent information contained in each part of the sentence is added to the new CLS vector. In summary, the steps in this section can be outlined as follows:

1. Integrate the influence of essential words into all other words.
2. Retrieve updated embeddings for all words as W′.
3. Compute the average of all embeddings, including CLS, to generate $\widehat{CLS}$.
4. Obtain the new CLS vector, $\widehat{CLS}$, which contains all information about the sentence meaning as well as the fine-grained influence of all word embeddings.

### Cognitive graph construction

**Fig. 2:**
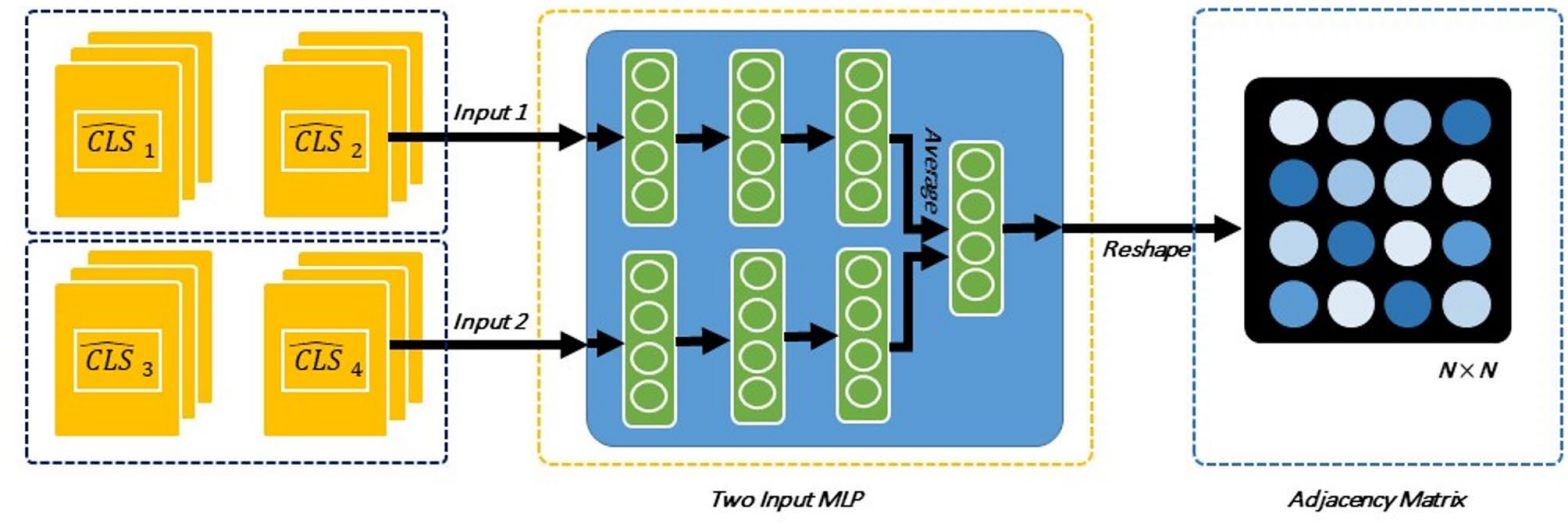
Creating the adjacency matrix from $\widehat{CLS}$ for edges of GAT with a two-branch MLP.

With $\widehat{CLS}$ after retrieving and aggregating all information, the need for a heterogeneous representation in the subsequent graph-based architecture is eliminated. To integrate the extracted embeddings with personality features, subjects and their personality information are modeled as a graph. This graph-based model uses vector representations for each node and aggregates neighborhood information, thereby capturing mutual influence and propagating information across node pairs. To overcome the complexity of constructing a heterogeneous graph, the previous steps were designed to enable the creation of a graph $G(V,E)$ with node features $V$ and edges $E$. The graph illustrates the relationship between words in a document and the personality traits of individuals, constructed based on both subject personality features and the semantic features of their papers. The initial embeddings, obtained from BERT, represent latent knowledge in the papers; therefore, positional encoding of words is implicitly applied via the vector embeddings. To account for the affective richness of each word and integrate it with overall word semantics, a single-way attention mechanism was designed in the previous stage. Accordingly, the new CLS vector, $\widehat{CLS}$, After applying the single-way attention mechanism, it is treated as shown in Fig. 1, where each node represents a subject. Next, the connectivity of graph nodes must be defined. Nodes are connected by undirected edges that indicate the similarity between two nodes. Initially, we considered something like the Pearson correlation between $\widehat{CLS}$ vectors and all important words for two distinct nodes. However, since node relationships cannot be modeled linearly, we devised a dynamic, task-specific approach to connect node pairs. To process each node independently and determine whether an edge may exist between nodes, a multi-branch approach is employed using $\widehat{CLS}$ vectors as input. The proposed method uses a two-branch multilayer perceptron (MLP) architecture, as shown in Fig. 2, where the $\widehat{CLS}$ the vector serves as input for all possible node pairings. Using this learning-based approach, edges can be dynamically learned according to the personality trait prediction task. It should be noted that $\widehat{CLS}$ contains information from multiple aspects, summarized as follows:

- Extracted using domain-specific fine-tuning of BERT.
- Encodes multi-level knowledge due to BERT’s multi-head self-attention layers.
- Implicitly reflects the affective richness of words through the single-way attention mechanism.

The adjacency matrix for representing the edges between nodes is computed as follows:

4 $$Adj=MLP\left({Branch}_{1}\right(\widehat{CLS}\left)\right|{Branch}_{2}\left(\widehat{CLS}\right))$$

**Fig. 3:**
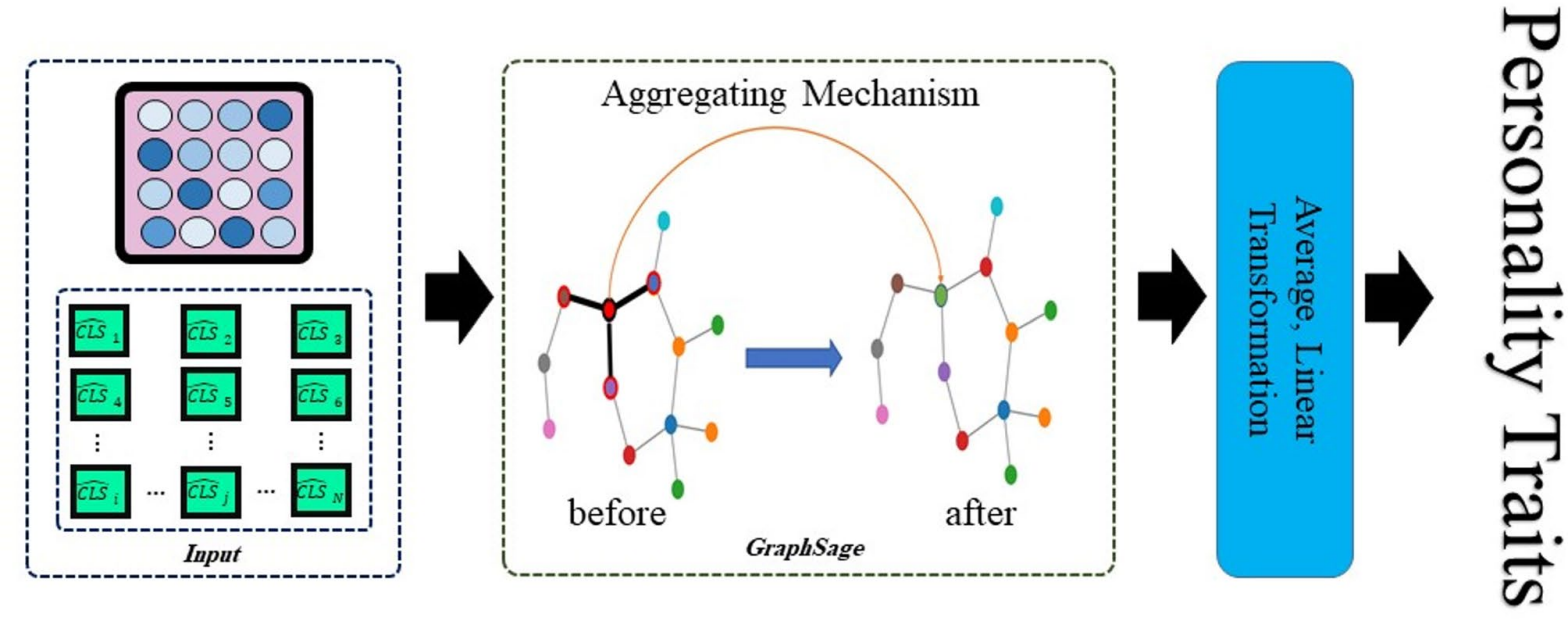
Graph network for classification of personality traits.

Here, $Adj \in R^{{D \times D}}$, and | denotes the concatenation operation. Two fully connected layers are used for each branch, and their outputs are combined through a merging layer to form a single vector, as shown in Fig. 2. After applying the sigmoid function, the dimensions are reshaped to obtain a ${\mathrm{D}} \times {\mathrm{D}}$ matrix, which serves as the adjacency matrix for the graph $G(V,E)$. Once the nodes and edges of the graph $G$ are defined, GraphSage can be used as the graph model^36^. GraphSage is an algorithm that samples neighboring nodes and aggregates their features, generating efficient representations without requiring the entire graph. These representations can be used for tasks such as node classification, link prediction, and clustering (Fig. 3). The representation of each node is updated by aggregating information from its neighbors, as shown in Eq. (5):

5 $$ \widehat{{{\mathrm{CLS}}}}_{{u_{u}^{{l + 1}} }}  = \sigma (W^{l} .(\widehat{{{\mathrm{CLS}}}}_{u}^{{\left( l \right)}} concatmean(W_{v} \sigma (\widehat{{CLS}}_{v}^{{\left( l \right)}} )\forall V(u))) $$

where ${\widehat{\mathrm{C}\mathrm{L}\mathrm{S}}}_{u}$ denotes the vector representation of node u at layer l, σ is an activation function introducing non-linearity, ${W}^{l}$ is a trainable matrix at layer l, ${W}_{v}$​ is another trainable matrix for neighboring nodes, and $V\left(u\right)$ is the subset of nodes in the neighborhood of node $u$.

### Output and prediction

The graph $G(V,E)$ is constructed using the $\widehat{CLS}$ embeddings as node features, with each feature vector corresponding to a subject in the dataset. This graph is designed for personality trait classification, where updated features at each layer can potentially enhance the overall architecture’s final performance. The input to the graph $G$ is considered as a set of node features, $\widehat{CLS}=\{{\widehat{CLS}}_{1},{\widehat{CLS}}_{2},\dots,{\widehat{CLS}}_{N}\}$ and ${\widehat{CLS}}_{i}\in{R}^{F}$ where N and F denote the number of nodes and the feature dimension of each node, respectively, each consecutive layer does not alter the dimension of the node features. To learn richer semantic knowledge, a linear transformation with learnable weights $W^{G}  \in R^{{F^{\prime }  \times F}}$ is applied to each node. The final graph embeddings are considered as the knowledge representations of each subject. Finally, a linear transformation is applied to the aggregated features, followed by a sigmoid function, to predict the personality traits.

In this method, binary cross-entropy is used, and the loss function between the ground-truth values and the predicted values is defined as follows:

6 $$Sigmoid\left(x\right)=\frac{1}{(1+{e}^{-x})}$$

7 $$BCE=-\left(ylog\right(\widehat{y})+(1-y\left)log\right(1-\widehat{y})$$

where ${y}_{p}$ denotes the ground-truth label of the personality trait, and ${\widehat{y}}_{p}$​ represents the predicted probability value.

### Dataset

To train the proposed method in this study, two publicly available datasets were utilized. The Essays dataset comprises 2,468 anonymous texts, with authors labeled according to the Big Five questionnaire^37^. One entry in this dataset was erroneous; therefore, to implement the proposed method, this entry was removed, and the remaining 2,467 samples were evaluated. Additionally, the myPersonality dataset includes 9,917 text entries from 250 Facebook users who participated in psychological research by completing the Big-5 questionnaire, with their corresponding scores recorded^38^.

## Results and discussion

Based on related studies in this field, we selected $K$ consecutive posts per subject and set the maximum sequence length to 250 for the BERT model. The learning rate was set to 1e-3 with a batch size of 32. Early stopping was used if the loss did not decrease for five consecutive epochs. The data were preprocessed by removing URLs, symbols, stop words, and emojis, and by converting all text to lowercase. Table 1 reports the accuracy of related studies alongside our proposed model. Additional analyses were conducted on different components of our architecture. According to Table 1, a comparison among recent studies on the Essays dataset shows that the proposed method achieved an average accuracy of 0.8063, demonstrating performance very close to that of study^30^ despite having a less complex architecture and outperforming study^34^. Table 2 compares the proposed model with related studies on the myPersonality dataset, demonstrating that it effectively extracts relevant knowledge about the target subjects. Furthermore, the results indicate that feature extraction using the single-way attention mechanism can improve model performance, especially compared with studies that did not explicitly integrate attention mechanisms into their architectures. Clearly, on this dataset, our method outperforms other related algorithms and achieves results very close to those reported in the related work. It is worth noting that the superior performance of the study^39^ on OPN (Openness to Experience) or CON (Conscientiousness) may be attributed to the deep features extracted from various hybrid models proposed by its authors. Studies employing transformer-based architectures or graph-based algorithms have shown promising results, as they can extract knowledge from information relevant to the target task. Extracting AGR (Agreeableness) trait features is not straightforward, possibly because individuals with AGR characteristics do not express them as explicitly in their writing. In contrast, other personality traits, such as NEU (Neuroticism) and EXT (Extraversion), can be directly conveyed.

**Table 1:** Comparison of the accuracy of the proposed method and related studies on the Essays dataset.

Model	Dataset	AGR	OPN	CON	NEU	EXT
Majumder et al.^19^	Essays	0.5671	0.6268	0.5730	0.5938	0.5809
Wung et al.^40^		0.5770	0.6480	0.5910	0.6300	0.6000
Ramezani et al.^41^		0.6031	0.5630	0.5918	0.6114	0.6425
Kerz et al.^42^		0.6016	0.7195	0.6138	0.6098	0.6301
Ren et al. ^34^		0.8030	0.8035	0.8023	0.8014	0.7994
Zhu et al. ^30^		0.807	0.818	0.796	0.817	0.811
Proposed method		0.7710	0.7954	0.8010	0.8217	0.8246

**Table 2:** Comparison of the accuracy of the proposed method and related studies on the myPersonality dataset.

Model	Dataset	AGR	OPN	CON	NEU	EXT
Wang et al.^40^	myPersonality	0.6800	0.8000	0.7600	0.7900	0.8000
Guerrero et al.^39^		0.6926	0.8136	0.7052	0.7254	0.6977
Zhu et al.^30^		0.727	0.823	0.826	0.827	0.825
Proposed Method		0.7115	0.8063	0.8199	0.8310	0.8289

To evaluate the effectiveness of the proposed method, detailed analyses were conducted on various components of the architecture. Specifically, we examined the impact of the threshold for highlighting important words, the single-way attention mechanism, the graph-based neural network, edge connections, and the sensitivity to specific key parameters in the proposed model. The importance of the single-way attention mechanism is confirmed by the results in Tables 3 and 4. This mechanism captures the influence of highlighted important words on other words and then reflects the updated word embeddings onto the CLS vector to obtain $\widehat{CLS}$ through averaging. The proposed mechanism takes the latent information from the highlighted words and implicitly propagates it to the CLS vector. In the single-way attention mechanism, the importance of key phrases is first applied to ordinary words, followed by averaging all embeddings, including CLS, to generate $\widehat{CLS}$. As shown in Tables 3 and 4, the overall effect of the single-way attention mechanism is positive, except for the CON trait in the Essays dataset, where its absence may lead to better performance. This outcome may be due to averaging embeddings negatively affecting the CLS embedding produced by the BERT model.

**Table 3:** Accuracy results for different configurations of the Single-way-attention mechanism on the Essays dataset.

State	Dataset	AGR	OPN	CON	NEU	EXT
Without single-way-attention	Essays	0.7222	0.7847	0.8113	0.7416	0.7597
Single-way-attention		0.7710	0.7954	0.8010	0.8217	0.8246

**Table 4:** Accuracy results for different configurations of the single-way attention mechanism on the myPersonality dataset.

State	Dataset	AGR	OPN	CON	NEU	EXT
Without single-way-attention	myPersonality	0.6662	0.7649	0.7575	0.7804	0.7433
Single-way-attention		0.7115	0.8063	0.8199	0.8310	0.8289

In this study, to compare and evaluate our choices, the well-known Graph Convolutional Network (GCN)^43^ was also compared, as shown in Tables 5 and 6. The results indicate that GraphSage with the proposed configuration achieved optimal performance. Since the overall architectures of GraphSage and GCN are very similar, similar performance is observed for the OPN trait on the Essays dataset and the NEU trait on the myPersonality dataset.

**Table 5:** Accuracy results of different graph architectures on the essays dataset.

Model	Dataset	AGR	OPN	CON	NEU	EXT
GCN [44]	Essays	0.7478	0.7812	0.7300	0.7619	0.7057
GraphSage [45]		0.7710	0.7954	0.8010	0.8217	0.8246

**Table 6:** Accuracy results of different graph architectures on the myPersonality dataset.

Model	Dataset	AGR	OPN	CON	NEU	EXT
GCN^44^	myPersonality	0.6848	0.7724	0.7012	0.8111	0.8072
GraphSage^45^		0.7115	0.8063	0.8199	0.8310	0.8289

**Fig. 4:**
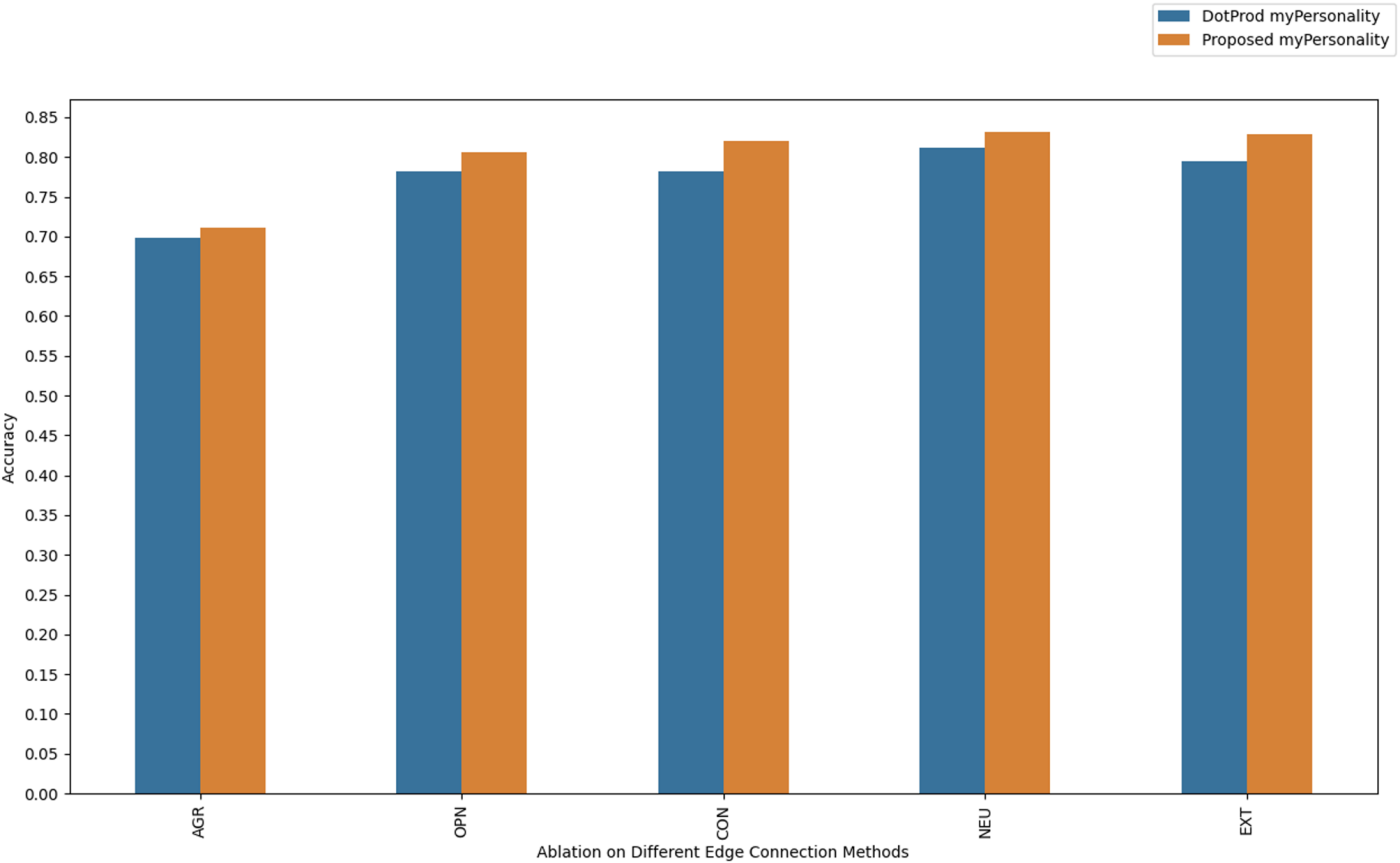
Performance comparison between the dot-product with thresholding and our proposed method for edge connection on the myPersonality dataset.

Next, the performance of the adopted edge connection method was evaluated on the myPersonality dataset, and an alternative edge connection method was also assessed. In this regard, the connection between two nodes could be computed using the dot product of the $\widehat{CLS}$ vectors of the two nodes along with a threshold. However, as shown in Fig. 4, the adopted edge connection method performs better. Although the proposed method demonstrates superior performance, the dot product approach with a threshold can still achieve relatively acceptable results.

**Fig. 5:**
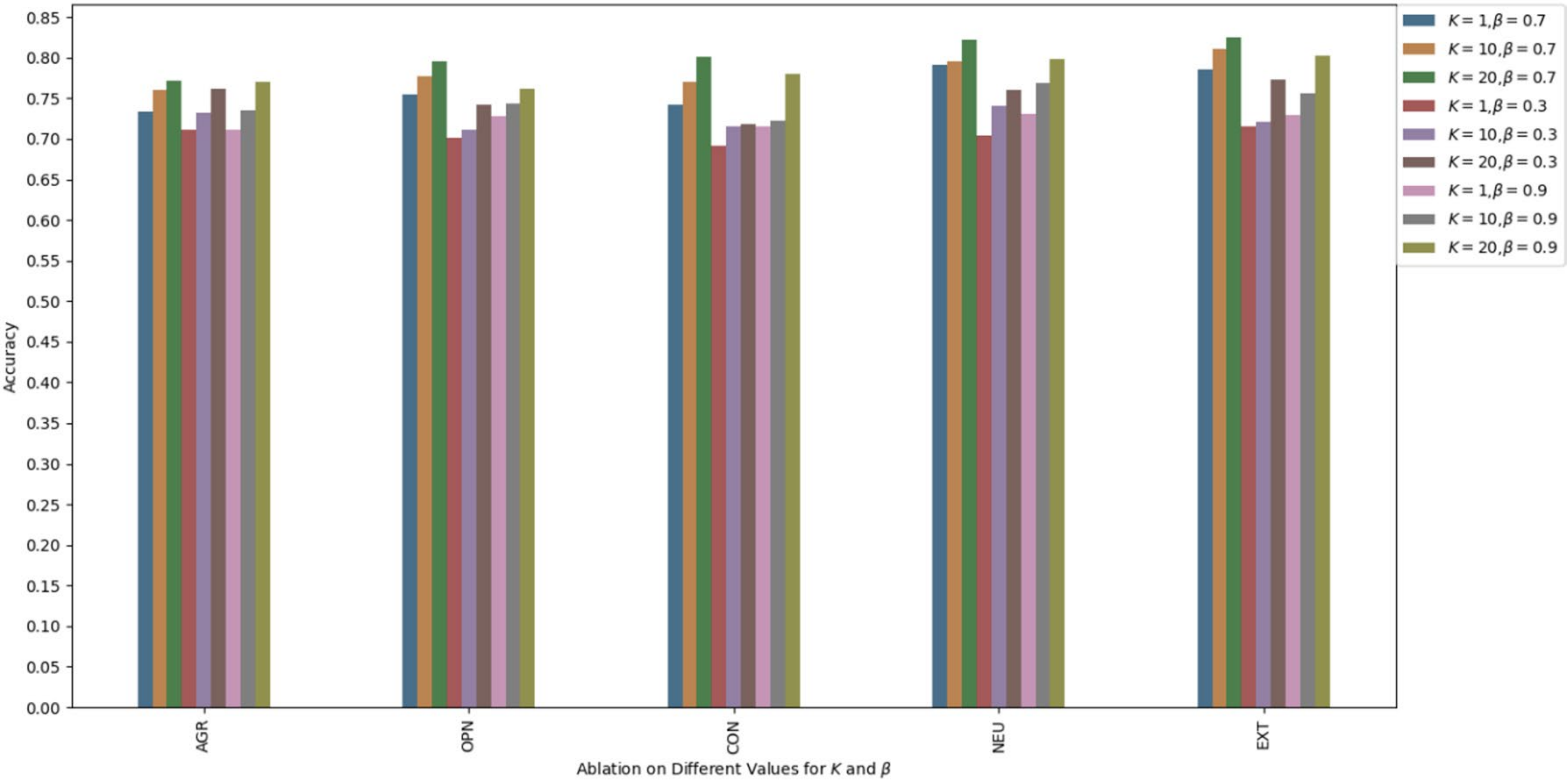
Analysis of different values of $\boldsymbol{K}$ and$\boldsymbol{\beta}$ on the Essays dataset.

To evaluate the impact of various parameters on personality detection, a series of experiments was conducted on key parameters, as shown in Fig. 5. This provides some justification for the effectiveness of the selected parameters. Based on the results in Fig. 5, three main observations can be inferred and are explained in detail as follows:

- The effect of increasing $K$ is always positive.
- Extremely low or high values of $\beta$ can negatively affect the output performance.
- The impact of $\beta$ is more dominant than K, implicitly confirming the importance of the single-way attention mechanism.

It is evident that the best performance across all personality traits is achieved with $K=20$ and β = 0.7. This suggests that applying K posts per subject can enhance the model’s performance, as aggregating information across multiple related posts can yield better embeddings for each node. The influence of $\beta$ highlighting important words is also illustrated in Fig. 5. It can be inferred that decreasing $\beta$ increases the number of highlighted important words, reducing the actual impact of enriched emotional words, while increasing $\beta$ may result in very few highlighted words, leading to suboptimal CLS embeddings and graph attention network edge connections. Additionally, the effect of β is much more pronounced than that of the $K$ parameter. Although $K$ can influence the results, the overall performance of the proposed architecture is more sensitive to changes in β.

**Fig. 6:**
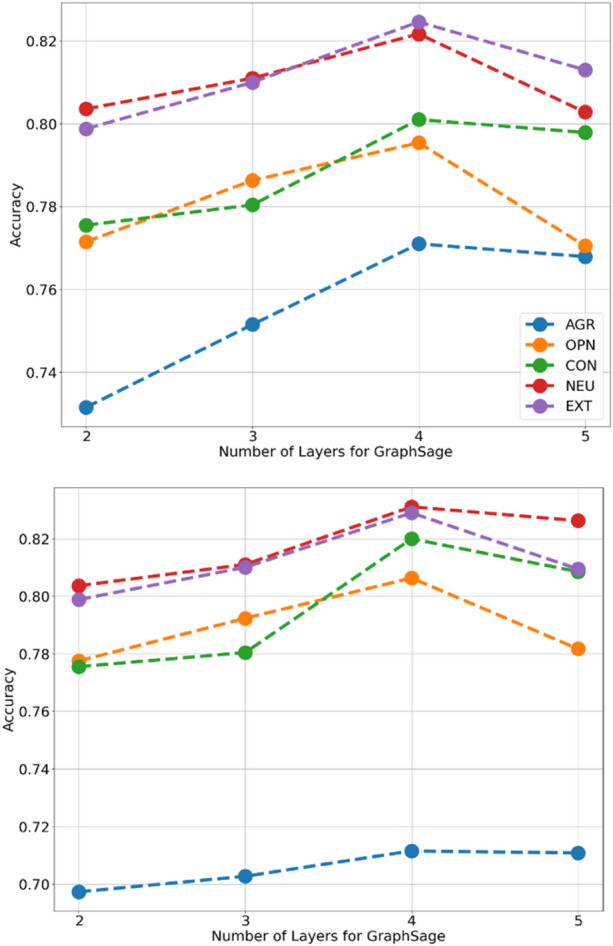
Effect of different GraphSage layers on the datasets: top, Essays; bottom, myPersonality.

Regarding the graph-based architecture configuration, the performance results for different numbers of GraphSage layers are shown in Fig. 6. Figure 6 shows that, in almost all cases, the architecture’s final performance is optimal with four layers. It is important to note that increasing the number of layers does not necessarily improve the final performance. The primary reason is that while additional layers enrich node embeddings, globally connecting each node to the entire graph can lead to node oversmoothing.

## Conclusion

In this study, we proposed a BERT- and GraphSage-based architecture for personality prediction. Our proposed model utilizes a domain-specific fine-tuned BERT for feature extraction. Additionally, using SenticNet, emotionally charged words were highlighted. The single-way attention mechanism integrates the impact of essential words with all other words, after which the average of all embeddings, including CLS, is considered to create $\widehat{CLS}$. The graph $G(V,E)$ is constructed using node features V and edges E. We treated the $\widehat{CLS}$ vector as node features, where each node represents a subject. Using a neural network-based method with a dynamic, task-related approach, we established connections between node pairs. To build the knowledge graph, we employed GraphSage, a graph-based model, and subsequently used it for personality trait prediction. Empirical results, ablation studies, and comparisons with other studies on the myPersonality and Essays datasets demonstrate the effectiveness and impact of the proposed model. Among the limitations of this study is the relatively small amount of available data. Technical challenges, such as the need for optimized hyperparameters, explainability, and generalizability, can also be considered limitations. Although hyperparameters were chosen based on previous studies and task-related considerations, and several detailed analyses were conducted for each, a separate investigation of each is still recommended. It should be noted that this approach requires high-performance hardware and is time-consuming. In the future, multimodal approaches incorporating audio, images, and text could further enhance knowledge extraction for more precise predictive system design. Beyond the general prospect of multimodal integration, several immediate extensions arise directly from our current limitations. One direction is to develop a more adaptive strategy for detecting salient cognitive or affective cues, allowing the model to dynamically adjust its notion of importance based on context rather than relying solely on predefined lexicons. Another promising extension is to investigate alternative cognitive or affective knowledge bases to evaluate how different sources influence the graph topology and downstream performance. Additionally, refining the graph construction process—such as incorporating variable edge weighting or discourse-level relations—could further enhance the model’s ability to capture nuanced psychological patterns in text. Future work will include visualization of attention patterns, analysis of salient nodes and edges, and other interpretability techniques to more clearly illustrate how the Cognitive Graph Transformer derives its predictions. Also we plan to conduct more comprehensive solvability analyses, including convergence behavior, optimization stability, sensitivity to initialization, and scalability evaluations across larger datasets and training configurations.

## Data Availability

The Essays dataset^31^ and the myPersonality dataset^32^, along with the proposed algorithm’s Python code, will be made available upon request to the relevant institution and will require authorization from the corresponding author.
